# Influence of co-creation signals on observers’ co-creation willingness: A self-determination theory perspective

**DOI:** 10.3389/fpsyg.2022.943704

**Published:** 2022-09-02

**Authors:** Li Zhang, Na Zhu, Hui Wang

**Affiliations:** Business School, Xiangtan University, Xiangtan, China

**Keywords:** co-creation signals, self-determination, co-creation willingness, mental simulation, signaling theory

## Abstract

With the service-dominant logic gradually replacing the traditional commodity-dominant reason, co-creating value with consumers has become an essential marketing practice for enterprises. As a critical information carrier in enterprise marketing communication, the co-creation signal impacts co-creation observers. Enterprises are now exploring how to effectively release co-creation signals to attract most observers to participate in value creation activities actively. Based on self-determination theory, this study investigates the influence mechanism of co-creation signals on observers’ willingness to co-create and the moderating role of mental simulation through the experimental method. The conclusions are as follows: (a) The co-creation signal has a significant positive effect on the co-creation intention of observers. Self-designed co-creation signals generate more important co-creation willingness than self-produced co-creation signals; (b) Self-determination and its dimensions mediate the relationship between co-creation signals and the observer’s co-creation willingness; and (c) Mental simulation moderates the relationship between co-creation signal and self-determination. This study has important theoretical significance in enriching co-creation signals and mental simulation research. It also provides management implications for enterprises to release co-creation signals and optimize co-creation marketing practices effectively.

## Introduction

As the service-dominant logic gradually replaces the traditional commodity-dominant logic as the new paradigm of marketing theory (Vargo and Lusch, 2004), value creation has shifted from one-way creation to co-creation (Prahalad, 2000). The role of consumers has changed from exchangers and extractors of value to creators of value and shapers of an enterprise’s competitive advantage (Ramaswamy and Gouillart, 2010). Co-creating value with consumers has become an essential marketing practice for enterprises. It helps enterprises to improve their flexibility, grasp market dynamics quickly and accurately, and meet the market demand at a lower cost (Reypens et al., 2016). In addition, co-creation can improve the innovation ability of enterprises (Hamidi and Gharneh, 2017), and enhance the satisfaction and loyalty of co-creation subjects (Cossío-Silva et al., 2016), it can effectively maintain the competitiveness and vitality of enterprises (Vega-Vazquez et al., 2013).

Several enterprises have used co-creation signals in their marketing communications, emphasizing consumer involvement in creating value in their products. For example, when Lego sells products designed by consumers, it uses prominent labels on the product packaging stating “designed by Lego fans.” The phenomenon of enterprises signaling co-creation has important market implications (Moreau and Herd, 2010). It affects consumers who have already been involved in co-creation as well as those who have not yet participated in value co-creation. Compared with the few consumers who have experienced co-creation, most consumers are observers of value co-creation (Kristal et al., 2016). Enterprises are now exploring how to effectively use co-creation signals to actively attract most observers to participate in value creation activities.

Previous studies have focused on consumers who have already participated in co-creation and found that they often engage in co-creation to meet the needs of cognition, entertainment, social networking, economic interests, and so on (Nambisan and Baron, 2009; Zwass, 2010; Ha et al., 2015). In addition, situational factors will also affect consumers’ co-creation behavior, such as the marketing strategy of enterprises, technical and emotional support provided by enterprises, and the community environmental atmosphere (Liang et al., 2011; Vargo and Lusch, 2016; Harmeling et al., 2017; Kliestik et al., 2022b; Nica et al., 2022). There are relatively few studies on consumers who have not yet participated in co-creation, namely co-creation observers, the most extensive market component. A few related studies have discussed how observers evaluate enterprises and their market offerings when they learn that other consumers participate in co-creation activities (Schreier et al., 2012; Thompson and Malaviya, 2013). These studies regard co-creation signals as a reliable source of information that can help customers make assumptions when they are exposed to a co-creation (Thompson and Malaviya, 2013), and explore the effect of co-creation signals on cognition, attitudes, and behaviors of observers. We seek to explore these impacts in greater depth.

Most studies have explored the co-creation signals that empower consumers to design. They found that consumer self-design co-creation signals can enhance the observers’ perception of customer orientation and the innovation ability of enterprises, thereby stimulating positive brand attitudes and reinforcing positive behavioral intentions (Fuchs and Schreier, 2011; Meißner et al., 2017). It can stimulate the sincere personality perception of co-creation observers to the brand and enhance their recommend willingness (Van Dijk et al., 2014). At the same time, the consumer identity of co-creation participants can facilitate the observer’s perception of product innovativeness (Schreier et al., 2012). Interpretations of co-creation signals often focus on the similarity between the observer and the participating customer in terms of identity and image (Fuchs et al., 2013; Kristal et al., 2016). In fact, according to signal theory, as the positive information of products or services released by enterprises to consumers, the co-creation signals can weaken the information asymmetry between enterprises and consumers (Connelly et al., 2011; Wells et al., 2011). Consumers will screen and interpret signals according to their own needs, which will make them form judgments and evaluations of enterprises, products, or services, and make the final consumption decisions (Dodds et al., 1991; Hu et al., 2015). In addition to similarity, co-creation signals stimulate observers to produce other cognitions or emotions. Self-determination theory emphasizes that individual behavior tendency is influenced by internal basic needs and external environment (Deci and Ryan, 2000). By participating in co-creation, consumers can obtain economic, cognition, societal, and entertainment benefits, it satisfies the need for autonomy, competence, and belonging. The initiative of enterprises to release co-creation signals to the market shows that there is a good external environment for consumers to participate in co-creation. Consequently, it is expected that after taking full account of external support and their own needs, observers believe that participating in co-creation can meet their basic psychological needs, and will produce a strong sense of self-determination, which makes them more likely to participate in co-creation. In addition, observers are consumers who have not participated in co-creation activities and lack a co-creation experience. Therefore, after receiving the co-creation signal, observers will be more inclined to imagine and rehearse the process or result of their participation in co-creation through mental simulation, thereby forming a certain cognitive state, that is, a sense of self-determination. Process simulation and outcome simulation guide individuals to imagine events from different emphases, which will make individuals differ in cognition and emotion (Taylor et al., 1998; Pham and Taylor, 1999). Accordingly, based on the characteristics of co-creation observers, the current study considers mental simulation as a moderator to explore the boundary conditions of co-creation signals in affecting observers’ sense of self-determination.

To sum up, as co-creation observer is the most extensive component of the market, it is necessary to explore how to effectively attract observers to participate in co-creation. The co-creation signal is the critical information carrier in the marketing communication of enterprises, and it will affect the recipient’s cognition, attitude, and behavior. Therefore, this study explores the influence mechanism of co-creation signals on the observer’s co-creation willingness from the perspective of the sense of self-determination and investigates the moderation role of mental simulation as a marketing communication strategy in this process. The theoretical implication of this study are as follows: (a) This study enriches and improves the research on the co-creation outcome from the perspective of co-creation observers; (b) This study uses the self-determination theory as a basis to explore the influence mechanism and effect difference of co-creation signal types, it extends and deepens co-creation signals research; and (c) Based on the characteristics of co-creation observers, mental simulation is selected as the moderating variable, which is more targeted and deepens the boundary research of the co-creation signal effect. At the same time, it is intended that this study can provide a reference for enterprises to effectively release the signal of co-creation, strengthen the cognition of observers of co-creation, and actively attract observers to participate in co-creation activities.

## Theoretical background and hypotheses

### Co-creation signals

When customers are confronted with a co-creation product, the co-creation signal is a reliable source of information to make speculation (Thompson and Malaviya, 2013). The concept of co-creation signals has not been clearly defined in existing research. But it has been found that co-creation signals have the following characteristics: it is the information that can be perceived, which aims to make receivers participate in co-creation activities, needs to be transmitted through some channels, and the creator can be either the company or the customer. Combined with the research purpose, the co-creation signal is defined as a set of information released by enterprises to make customers perceive and participate in value creation activities. Customers can participate in value co-creation at various stages of value generation, such as concept formation, self-design, self-production, and self-logistics (Payne et al., 2008). However, due to the limitations of value co-creation conditions and customers’ existing co-creation knowledge, customer participation in co-creation is currently concentrated in the design and production stages of the value chain. That is, it primarily involves self-designed and self-produced value co-creation (Etgar, 2008; Atakan et al., 2014). This study maintains that the co-creation signals emitted by companies are also mostly consumer self-designed co-creation signals and consumer self-produced co-creation signals.

Several studies have explored the effect of co-creating signals from an observer’s perspective. When enterprises release signals of customer participation in product co-creation, it can stimulate positive attitudes, perceptions that the brand is customer-oriented, and perceptions of brand sincerity among observers. These perceptions and attitudes trigger positive behavioral intentions, such as an increased trial, repeat purchase, positive word-of-mouth and willingness to recommend (Fuchs and Schreier, 2011; Van Dijk et al., 2014). When the company sends out the signal of customer participation in product selection, the product can appeal to a broader consumer group. It can stimulate observers’ perceptions of the enterprise’s innovative capabilities, leading to positive behavioral intentions, such as positive word-of-mouth and purchase intentions (Meißner et al., 2017).

In advertising co-creation, some research has found that when the advertisement provides the background information of customers who have participated in the advertising co-creation, and observers review advertising information with limited cognitive resources. Observers can effectively enhance similarity perception, and generate higher advertising evaluation and brand evaluation (Thompson and Malaviya, 2013). However, co-creation signals do not always have a positive effect. Some studies have found that the valence of customer engagement depends on the consumer’s cognitive interpretation of the co-creation signal. If the signals are positively interpreted, positive outcomes such as service co-creation are expected, but if they are negatively interpreted, negative outcomes such as service co-destruction are predicted (Siddique et al., 2021). For some complex products, ordinary consumers may lack sufficient professional knowledge and ability to participate in value creation, and the transmission of co-creation signals will make observers skeptical about innovation (Franke et al., 2009; Schreier et al., 2012). Research on luxury products further shows that consumer design co-creation signals reduce the observer’s perception of product quality, and weaken the symbol of product identity or status (Fuchs et al., 2013), thus it is difficult to enhance observers’ brand equity (Kristal et al., 2016).

In summary, co-creation signals as an environmental stimulus can influence customers’ attitudes and behaviors. A few scholars have researched the effects of co-creation signals on observers. Still, fewer studies have clearly distinguished between the types of co-creation signals and the differences in their impact and have focused on similarity when investigating the mechanisms of influence.

### Sense of self-determination

The Sense of self-determination is derived from the theory of basic psychological needs, a core sub-theory of self-determination theory. It suggests that individuals are born with three basic psychological needs: autonomy, competence and belonging (Deci and Ryan, 2008), the individual’s satisfaction with the above three psychological needs forms a sense of self-determination. It can be divided into three dimensions: sense of autonomy, sense of competence, and sense of belonging (Deci and Ryan, 2000, 2013).

Sense of autonomy refers to the perception of the degree to which individuals can dominate their behavior and make choices freely with full awareness of environmental conditions (Dahl and Moreau, 2007), emphasizing a sense of independence and control. Sense of competence is the individual’s perception of the effectiveness of their behavior and whether they can achieve desired goals, which is the individual’s subjective assessment of their competence (Ryan, 1995). Sense of belonging is the individual’s subjective perception of the extent to which they are interconnected with others and are cared for, respected and needed by others (Hsieh and Chang, 2016).

The individual’s sense of self-determination will be affected by the external environment. The self-supporting organizational environment and management style can meet the basic psychological needs of employees for autonomy, competence, and belonging. Employees will generate a higher sense of self-determination, and strengthen their internal work motivation (Deci et al., 2001; Chirkov et al., 2003). In the process of interaction between enterprises and consumers, enterprises create a supportive environment that provides consumers with sufficient resources and training, which helps to build a stable relationship foundation and enhance consumers’ sense of competence (McKee et al., 2006; Rosenbaum et al., 2007). In the marketing field, research has chiefly explored the impact of self-determination on consumer perceptions, emotions, and behaviors. In online communities, consumers with a higher sense of self-victory and community belonging are more likely to participate in community activities and have contribution behaviors such as continuous sharing of experience and professional knowledge (Hsu et al., 2007; Chai and Kim, 2012; Chiu et al., 2015). When the needs of autonomy, competence, and belonging are met, consumers will have higher brand attachment and community attachment (Chou and Yuan, 2015). Value co-creation emphasizes the customer’s dominant position and is a hot subfield of self-determination research. Extrinsic and intrinsic motivations of self-determination drive individuals to participate in value co-creation (Osei-Frimpong, 2017). Sense of self-determination can affect an individual’s organizational embedding intention and identity. High identification and high embedding intention make the individual more likely to co-create value in interactions with the brand (Greguras and Diefendorff, 2009). In summary, external environmental factors can impact individuals’ self-determination, influencing their cognition and behavior. Therefore, the Sense of self-determination is often seen as a mediating variable to explain the specific processes that generate positive consumer brand behaviors.

### Mental simulation

Mental simulation is a human mental activity generated spontaneously by the individual or under the stimulation of external situational factors (McConnell et al., 2000). It has two main functions: problem-solving and emotion management (Taylor et al., 1998). As a simulated mental representation, mental simulation is the individual’s imagination or recollection of the process or outcome of an event or series of events, including the rehearsal of a possible future event, the reconstruction of an occurred event, the imagination of an event, and the combination of natural and imagined events (Taylor and Schneider, 1989). According to the thinking orientation, mental simulation is most commonly divided into process and outcome simulation. Process simulation guides individuals to recall or imagine specific processes and steps to achieve goals. It helps to formulate effective plans and increase the likelihood of achieving goals. Outcome simulation leads individuals to recall or imagine the impact of outcomes, usually the desired outcome, and can encourage individuals to work toward their desired outcome (Taylor et al., 1998; Pham and Taylor, 1999; Escalas and Luce, 2003).

Scholars have mostly explored the differential effects of mental simulations on purchase intentions from different research perspectives. It has been found that mental simulation can promote the connection between cognitive activities and actual behaviors (Taylor and Schneider, 1989), positively changing individuals’ attitudes, behavioral intentions, or basic actions (Escalas, 2004). For example, compared with the outcome simulation, strong advertising words make consumers of process simulations more receptive to advertisements, leading to stronger purchase intentions (Escalas and Luce, 2003).

The utility of mental simulation varies across different information processing modes. In the cognitive model, only outcome simulation can significantly promote consumers’ purchase intention, while in the affective mode, only process simulation can strongly motivate consumers to buy (Zhao et al., 2011). Mental simulation has also been widely used in new product marketing research. When faced with new products, consumers often spontaneously generate relevant images through mental simulation to recognize and evaluate the new product (Hoeffler, 2003). In addition, mental simulation can help consumers learn and understand new products, reduce perceptions of uncertainty, and thus enhance their assessment and purchase intentions about new products (Zhao et al., 2014). Overall, existing studies have mainly discussed the effects of mental simulation on purchase intention and product evaluation. However, there is still much room for further research on mental simulation as a moderating variable.

### Co-creation signals and observers’ co-creation willingness

Signaling theory suggests that information about products available to enterprises and consumers is asymmetric in market transactions. Companies can reduce this asymmetry by actively signaling positive information about products or services to consumers, influencing their attitudes and behaviors (Connelly et al., 2011; Wells et al., 2011). A co-creation signal is a message that the enterprise releases co-creation activities to the market, emphasizing that some consumers have participated in co-creation activities. Such signals aim to induce more consumers to participate in co-creation actively. This signal also acts on consumers who have participated in co-creation activities to enhance their enthusiasm for participation. More importantly, consumers who have not yet participated in co-creation can be affected by the signal of co-creation of the brand. Accordingly, they will feel the customer-centered service orientation of the enterprise. In addition, such activities will affect their perception of product and service quality and enhance the observers’ attention, trust, and recognition of the company.

Product co-creation research finds that enterprises with authorized consumers designing and choosing products can make observers perceive stronger customer orientation than zero-licensing enterprises (Fuchs and Schreier, 2011). In addition, when an enterprise shows that its products are co-created by consumers, the consumers will increase their awareness of the enterprise’s innovation capability and product innovation (Schreier et al., 2012; Meißner et al., 2017). After interpreting such information from co-creation signals, observers think that the enterprises and brands involved in co-creation are more truthful and sincere. They will thus form a positive brand attitude, which reinforces their positive behavioral intention. These positive behavioral intentions include repeat purchase, brand identification, brand loyalty, positive word-of-mouth and recommendation behavior (Fuchs and Schreier, 2011; Van Dijk et al., 2014). With this logic, this study suggests that observers are most likely to make a series of positive associations and intentions after receiving the co-creation signal from the company. In addition, positive results occur regardless of whether consumers self-produced co-creation signals or self-designed co-creation signals.

However, studies have shown that self-produced co-creation and self-designed co-creation lead to different product evaluations by consumers (Buechel and Janiszewski, 2014). Observers who have received the corresponding signals, resulting in various positive associations, also have positive behavioral intentions. When receiving the indication of self-designed co-creation, observers will interpret it as giving consumers more freedom, access to technology, and challenges. Consumers mainly conceive and create products or services they need through knowledge or mental labor input, which displays stronger design innovation. Design results can more effectively express their values, personalities, and preferences (Franke et al., 2010). Observers will experience a high degree of product personalization, solid psychological connections, and emotional attachments when imagining participating in self-designed co-creation. Therefore, they will have a stronger intention to co-create.

Observers who receive the self-produced signal find that co-creation allows them to participate in the process of manufacturing, assembling, or reconfiguring. This participation in skills and other manual labor leads to a stable operation structure that produces more standardized products. Consumers are only involved in the consumption-based processing of products (Troye and Supphellen, 2012), and cannot integrate their beliefs and preferences into products. Relevant machine learning research also shows that perceived personalization considerably predicts co-creation value intention (Hopkins, 2022; Kliestik et al., 2022a). Therefore, observers do not necessarily have significantly stronger co-creation willingness after receiving self-produced co-creation signals.

*H1*: Co-creation signals has a significant effect on observers’ co-creation willingness, and it is expected that self-designed co-creation signals generate a more vital willingness to co-create than self-produced co-creation signals.

### Co-creation signals and sense of self-determination

According to self-determination theory, individuals have a self-determined tendency to direct their self-development and internal growth and are influenced by the external environment. Individuals exhibit positive behavior when the external environment satisfies three basic psychological needs (Deci and Ryan, 2000). By engaging in co-creation, consumers can reap economic, cognitive, social, and recreational benefits. They can even nurture or enhance self-confidence, creativity, and a sense of responsibility (Nambisan and Baron, 2009, 2010).

When an enterprise actively uses co-creation signals to the market, it hopes more consumers will participate in co-creation activities. At the same time, it also means that enterprises will provide specific emotional support or operational support in co-creation. Co-creation signals make observers aware that other consumers are involved in co-creation activities, reducing the observers’ uncertainty and risk perception about co-creation. Therefore, participation in co-creation is consistent with observers’ internal tendency to pursue self-development and self-integration. The transmission of co-creation signals from companies indicates a favorable external environment for observers to participate in co-creation. After fully considering the external environment and internal needs, observers believe that participation in co-creation can satisfy their basic psychological needs, which leads to a strong sense of self-determination.

Firstly, in the process of co-creation, enterprises often respect co-creation participants, avoid excessive control and support the interactive process so that participants can master a certain degree of autonomy. Consumers can integrate their own beliefs, personalities and preferences into products (Atakan et al., 2014; Hsieh and Chang, 2016). Such co-created products often have benefits and innovations expected by consumers, enhancing participants’ sense of autonomy (Vargo and Lusch, 2004; Franke et al., 2006).

Secondly, enterprises encourage empowered and developmental service interactions in the process of co-creation, which is conducive to improving the perceived competence of co-creation participants (Karpen et al., 2015; Hsieh and Chang, 2016). In addition, consumers invest knowledge, experience, skills, and physical strength to participate in all aspects of product co-creation. It provides an opportunity for them to demonstrate their ability and helps strengthen consumers’ confidence, sense of competence, and sense of achievement (Hsu et al., 2007; Franke et al., 2010). The continuous enhancement of interaction and collaboration in the co-creation engagement process helps consumers enjoy the co-creation experience and enhance self-efficacy (Buechel and Janiszewski, 2014).

Finally, the co-creation by enterprises and consumers reflects the enterprise’s customer-centered service orientation. Consumers are more likely to perceive being cared for, respected, understood, and appreciated, to improve their perceived relatedness (Gagné, 2003). In the process of product co-creation, consumers have high-level interaction and cooperation with enterprises and other consumers, which makes it easier to establish a trust-based long-term partnership and foster a psychologically fair climate (Chiniara and Bentein, 2016). Enterprises create an environment conducive to the establishment of intimate relationships with consumers, to improve the perceived relatedness and perceived belonging of consumers (Karpen et al., 2015).

In product co-creation, self-designed co-creation by consumers requires more mental effort and professional skills. The co-creation process is full of freedom, expertise, and challenges (Buechel and Janiszewski, 2014). The designed product incorporates consumers’ beliefs, personalities and preferences, a combination of self-extension, achievement proof and identity expression. Moreover, consumers will have a stronger sense of creativity, autonomy, and pleasure during participation (Dahl and Moreau, 2007). At the same time, the high degree of participation and strong interaction of consumers in self-designed co-creation will make them have a strong psychological connection and emotional attachment to personalized design achievements and co-creation enterprises (Franke et al., 2010; Atakan et al., 2014), and then form a high sense of belonging.

Consumer self-produced co-creation is more about investing in basic skills or manual labor, and the co-creation products are still mostly standardized (Terblanche, 2014). Consumers involved in co-creation can acquire production achievements and process experience. The co-creation process is more structured and less risky, and consumers have relatively low autonomy and perceive less pleasure, achievement and efficacy, which leads to lower psychological attachment, psychological ownership, and belonging (Dahl and Moreau, 2007; Walasek et al., 2017). Accordingly, this study suggests that after receiving different types of co-creation signals, observers have a different sense of autonomy, competence and belonging when interpreting the signals and imagining their participation in product co-creation.

*H2*: Co-creation signals has a significant effect on observers’ sense of self-determination, and it is expected that self-designed co-creation signals generate a stronger sense of self-determination than self-produced co-creation signals.

*H2a*: Co-creation signals has a significant effect on observers’ sense of autonomy, and it is expected that self-designed co-creation signals generate a stronger sense of autonomy than self-produced co-creation signals.

*H2b*: Co-creation signals has a significant effect on observers’ sense of competence, and it is expected that self-designed co-creation signals generate a stronger sense of competence than self-produced co-creation signals.

*H2c*: Co-creation signals has a significant effect on observers’ sense of belonging, and it is expected that self-designed co-creation signals generate a stronger sense of belonging than self-produced co-creation signals.

### Mediating effect of self-determination

Self-determination theory emphasizes that when the three basic psychological needs of individuals are met, conditions are conducive to the formation of positive emotions such as happiness and are conducive to the internalization of individual external motivation, which positively affects behavior (Deci and Ryan, 2000). Individuals’ sense of autonomy and competence positively affects their experiences. This result is not affected by factors such as the type and duration of activities they are involved in (Downie et al., 2008). A positive experience will promote a sense of individual identity, thus forming a sense of belonging, enhancing personal support for their group, and increasing the degree of individual participation (Lyu, 2012). Co-creation studies have found that as a motivational factor, individuals’ knowledge self-efficacy have a significant impact on individual engagement in value co-creation through perceived benefits (Al-Kumaim et al., 2021). Self-determination can improve the embedding intention of individuals in organizations, prompting consumers to change from controlled motivation to funded motivation. Consumers are more likely to actively participate in value co-creation (Greguras and Diefendorff, 2009). When consumers perceive greater autonomy, competence, and belonging, they will exhibit more customer participation behavior. When consumers experience higher relevance, they will show more customer citizenship behavior (Li et al., 2021). Thus, individuals with a higher sense of self-determination are more likely to be intrinsically motivated to show a higher degree of interest and engagement in the task (Hsieh and Chang, 2016).

According to the S–O–R model, external stimuli can influence individuals’ perceptions and attitudes through mental activities and internal cognitive processes, and then individuals will generate corresponding behavioral responses (Namkung and Jang, 2010). Self-determination is a psychological, cognitive state formed by consumers considering the support of the external environment and their own internal needs to mediate the influence of external stimuli on consumer behavior. In this study, co-creation signals transmitted by enterprises belong to the external stimulus that conveys to the observers a customer-centered service orientation and calls for more consumers to participate in co-creation actively. In interpreting the signals and imagining their participation in co-creation, observers will have psychological cognition, that is, the sense of self-determination, ultimately affecting their willingness to participate.

*H3*: Observers’ sense of self-determination mediates the relationship between co-creation signals and observers’ co-creation willingness.

*H3a*: Observers’ sense of autonomy mediates the relationship between co-creation signals and observers’ co-creation willingness.

*H3b*: Observers’ sense of competence mediates the relationship between co-creation signals and observers’ co-creation willingness.

*H3c*: Observers’ sense of belonging mediates the relationship between co-creation signals and observers’ co-creation willingness.

### Moderating effect of mental simulation

There are specific prerequisites for the inspiration of mental simulation. Firstly, when consumers need to make decisions under uncertain conditions, they will rely more on the cognitive decision-making path based on mental simulation. Mental simulation’s problem focus and emotion management functions can provide a framework for consumers to solve problems, affecting their information processing results (Krishnamurthy and Sivaraman, 2002). Secondly, normality, variability of the premise, and consumers’ motivation are the main factors that affect the generation of mental simulation (Kahneman and Miller, 1986; Wells and Gavanski, 1989). Finally, external stimuli can initiate mental simulation through stories or information in narratives (Escalas, 2004). It can be speculated that consumer participation in co-creation deviates from the paradigm of company-independent value creation, leading to abnormalities. The popularity of customer-oriented service logic makes the premise of enterprise independent value creation highly variable.

At the same time, observers are consumers who have not yet participated in co-creation and lack experience. They have the motivation to understand anonymous information through mental simulation. Thus, when observers receive a co-creation signal from the enterprise, they are more inclined to interpret it through the relevant information displayed by the enterprise, combined with their limited understanding. Simultaneously, they will perform corresponding mental simulations to imagine the process or result of participating in the co-creation, thus forming a particular cognitive state, i.e., sense of self-determination.

Mental simulation can be divided into process and outcome simulation. Different mental simulation techniques can lead to cognitive and affective differences in individuals, influencing their future behavior. Research has found that process simulation can effectively increase individuals’ perceived control and subjective norms, thus changing their behavioral intentions toward an action (Armitage and Reidy, 2008). The research on advertising effect shows that, compared with outcome simulation, process simulation manipulates consumers into narrative thinking through powerful advertising words. It makes consumers more sensitive to advertising and more effective in the persuasion effect of advertising, thus making consumers have stronger purchase intention (Escalas and Luce, 2003; Escalas, 2004).

In evaluating new products, mental simulation of the product usage process can help consumers reduce uncertainty about new products and establish more stable preferences (Hoeffler, 2003). In addition, the effects of process and outcome simulations on product evaluation differ depending on the mode of information processing. Process simulation leads to higher positive cognition such as product evaluation in the cognition-dominated information processing mode. In the emotion-dominated information processing mode, outcome simulation brings higher product evaluation and other positive behaviors (Zhao et al., 2011).

The co-creation observers have not participated in the co-creation process and have no previous co-creation experience. Therefore, they are more inclined to interpret co-creation signals with a cognition-dominated information processing mode when receiving co-creation signals transmitted by enterprises. In this mode, compared to outcome simulations, process simulations can help observers develop concrete co-creative action plans. In this way, the uncertainty of co-creation is effectively reduced. The observers can experience autonomy in the process of imagining participating in co-creation, so that initiative and interaction can be brought into play. It involves a strong sense of determination as reflected in its three dimensions: autonomy, competence and belonging.

*H4*: Mental simulation moderates the relationship between co-creation signals and sense of self-determination, with observers performing process simulation generating a stronger sense of self-determination compared to outcome simulation.

*H4a*: Mental simulation moderates the relationship between co-creation signals and sense of autonomy, with observers performing process simulation generating a stronger sense of autonomy compared to outcome simulation.

*H4b*: Mental simulation moderates the relationship between co-creation signals and sense of competence, with observers performing process simulation generating a stronger sense of competence compared to outcome simulation.

*H4c*: Mental simulation moderates the relationship between co-creation signals and sense of belonging, with observers performing process simulation generating a stronger sense of belonging compared to outcome simulation.

**Figure 1:**
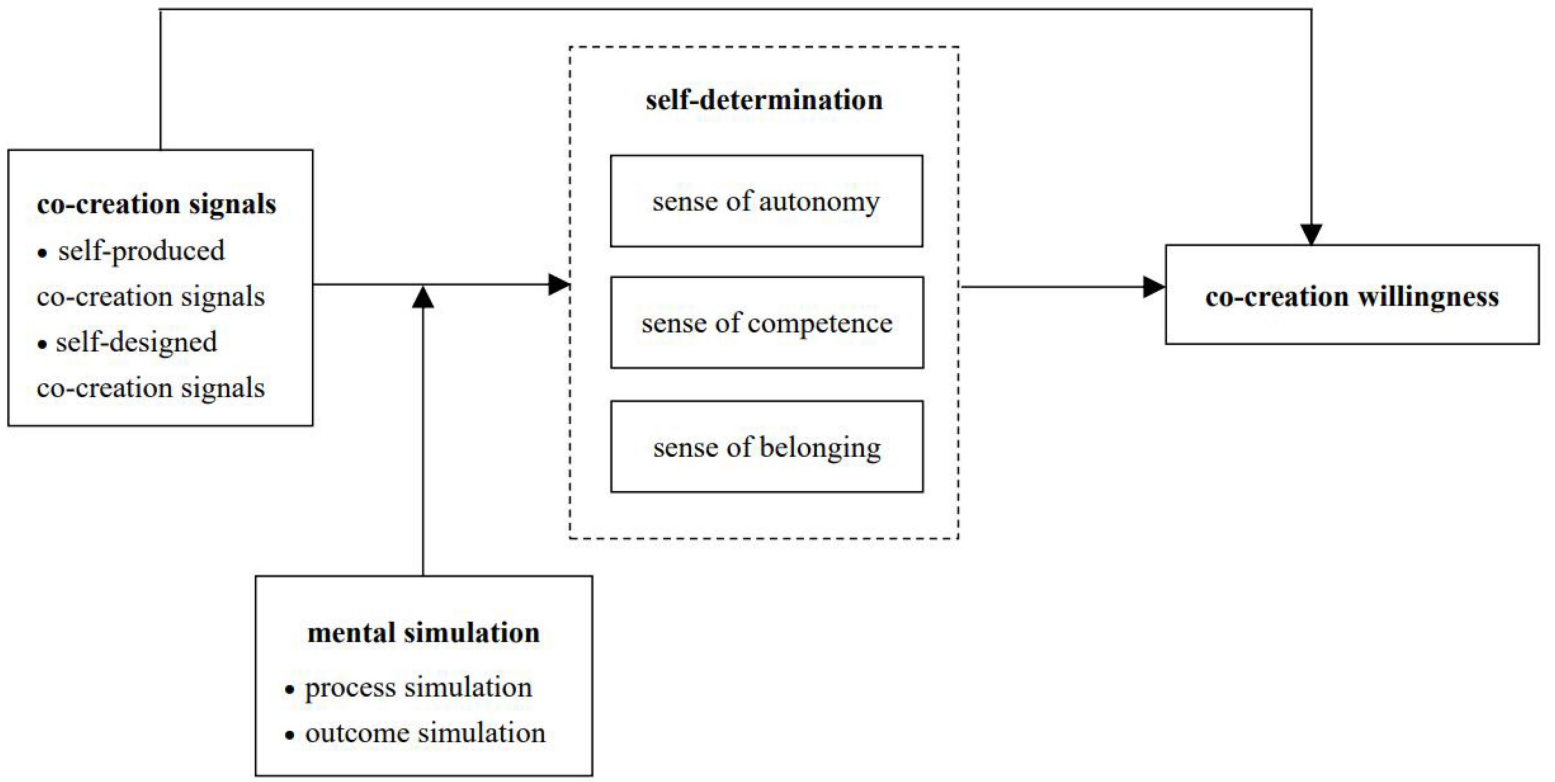
Theoretical model.

In summary, the theoretical model for this study is shown in Figure 1.

## Study 1: Main effects and mediating mechanisms of co-creation signals

### Pretest

The primary purpose of the pretest was to test the validity of experimental material, experimental context, and types of co-creation products. T-shirts are often used as an excellent experimental product in value co-creation research (Atakan et al., 2014). Because compared with other products, it can not only effectively reflect the participation of value co-creation, but also the process is simple, controllable, and inexpensive. So this experiment also used T-shirts as experimental stimuli. The stimuli were referred to as “Brand A T-shirts” to avoid the interference of specific brand associations. This experiment adopted a one-way between-group design. Subjects were randomly assigned to a consumer self-produced co-creation signal group, a consumer self-designed co-creation signal group and a control group. The first two groups showed the virtual news containing information on consumer involvement in the production or design of T-shirts. The control group was only briefly introduced to the craft of T-shirts in the virtual news. After the experiment, subjects in the two co-creative signal groups answered about co-creation signal type discrimination. Subsequently, the three groups filled in the 7-point Likert scale of co-creation willingness (Zwass, 2010). Finally, subjects’ essential demographic characteristics and experience in co-creation were investigated. The pretest recruited 104 students (49 male/55 female; mean age = 18.875 years old) from a large public university in central China in mid-August 2021.

The results of the pretest *t-test* showed that subjects can effectively determine the difference between self-produced co-creation signal and self-designed co-creation signal (*M*_produced_ = 2.059, *M*_designed_ = 5.583, *t* = −18.613, *p* < 0.05). A one-way ANOVA showed a significant difference in co-creation willingness among the three groups (*F* = 43.915, *p* < 0.05). After multiple LSD comparisons and *t*-test in pairs, it found that subjects in the self-produced co-creation signal group and the self-designed co-creation signal group had a stronger participation willingness than the control group (*M*_produced_ = 4.949, *M*_controlled_ = 4.184, *t* = 5.539, *p* < 0.05; *M*_designed_ = 5.417, *M*_controlled_ = 4.184, *t* = 9.214, *p* < 0.05), and subjects in the self-designed co-creation signal group had a more vital willingness to participate compared to the self-produced co-creation signal group (*M*_designed_ = 5.417, *M*_produced_ = 4.949, *t* = −3.670, *p* < 0.05). Accordingly, the pretest results confirmed that the co-creation signal type was successfully manipulated. The experimental material, experimental context and co-creation product type can be used in formal experiments. In addition, H1 has been preliminarily verified: co-creation signal has a significant effect on observers’ co-creation willingness. Self-designed co-creation signals lead to stronger co-creation willingness among observers than did self-produced co-creation signals.

### Experiment design

The primary purpose of experiment 1 was to examine the effects of different co-creation signal types on observers’ co-creation willingness and the mediating role of self-determination, i.e., to test H1–H3. The experiment adopted a one-way between-group design (self-produced co-creation signal vs. self-designed co-creation signal). Specifically, at the beginning of the experiment, two groups of subjects were assigned to click a web link, respectively, to browse the situational material. Among them, the situational material of the consumer self-produced co-creation signal group was “Q company invites consumers to its offline stores to experience the production of T-shirts. In the store, participants can use a small printing machine to print a designated pattern on a T-shirt, or they can take the form of hand-drawn. The T-shirts produced by consumers will be displayed in offline stores and on the company’s official website.” The situational material of the consumer self-designed co-creation signal group was “Q company solicits the design scheme of new T-shirts from consumers, and consumers can use their imagination to create. The company will select the ten best designs for production, and the design works will also be displayed in offline stores and on the company’s official website.” After reading the material, two groups of participants were asked to answer the co-creation signal type discrimination questions. The second part measured the participants’ sense of self-determination, the sense of autonomy scale, and the sense of competence scale drew on the study by Dahl and Moreau (2007), Hsieh and Chang (2016). The sense of belonging scale drew on the research of Nambisan and Baron (2009), each dimension using three items for measurement. In the third part, the participants were asked to fill in the co-creation willingness scale (Zwass, 2010), with a total of 4 items. All were 7-level Likert scales and were slightly modified according to the experimental context. The investigation recruited 184 participants at a large public university in central China in late September 2021, with 174 valid subjects (75 male/99 female; mean age = 18.759 years old).

### Experiment results

#### Scale reliability and validity

The statistical software SPSS 26.0 was used to test the reliability of the scales. The Cronbach’s *α* coefficients for the sense of autonomy, sense of competence, sense of belonging, and co-creation willingness were 0.888, 0.839, 0.845, and 0.849, respectively, which all reached the reliability standard required by statistics. Thus, the reliability of the overall scale was good. The statistical software AMOS 24.0 was used to conduct confirmatory factor analysis to test the validity of the scales. Firstly, the average variance extracted (AVE) of sense of autonomy, sense of competence, sense of belonging, and co-creation willingness, respectively, were 0.732, 0.641, 0.654, and 0.583, all of which were greater than the critical value 0.5 (Fornell and Larcker, 1981). The results indicated that the scale had good convergent validity. Secondly, CFA results also showed that compared with other competition models, the theoretical four-factor model fitted well with the data (*χ*^2^/*df* = 2.071, RMSEA = 0.079, CFI = 0.964, TLI = 0.953, IFI = 0.965; Table 1), indicating satisfactory discriminant validity. In conclusion, the scales in this study had good reliability and validity and were suitable for subsequent hypothesis testing.

**Table 1:** Confirmatory factor analysis results.

Model	*χ* ^2^	*df*	*χ*^2^/*df*	RMSEA	CFI	TLI	IFI
Four-factor model	122.170	59	2.071	0.079	0.964	0.953	0.965
Three-factor model (a)	177.863	62	2.869	0.104	0.935	0.918	0.935
Three-factor model (b)	153.267	62	2.472	0.092	0.949	0.935	0.949
Three-factor model (c)	164.696	62	2.656	0.098	0.942	0.927	0.943
Two-factor model	201.114	64	3.142	0.111	0.923	0.906	0.923
Single-factor model	246.010	65	3.785	0.127	0.878	0.898	0.899

#### Maneuverability test

*t*-Test was conducted on the judgment item data of co-creation signal type. The results showed that subjects could effectively judge the difference between self-produced co-creation signals and self-designed co-creation signals (*M*_designed_ = 6.000, *M*_produced_ = 1.930, *t* = −45.603, *p* < 0.05). The experimental manipulation of the co-creation signal was successful.

#### Main effects test

*t*-Test results of co-creation willingness suggested that there were significant differences in co-creation willingness after receiving different co-creation signals. Subjects in the self-designed co-creation signal group have stronger co-creation intentions than self-produced co-creation signal group (*M*_designed_ = 5.327, *M*_produced_ = 4.369, *t* = −12.763, *p* < 0.05). Compared to self-produced co-creation signals, observers who received self-designed co-creation signals reported that they will be more inclined to engage in co-creation, and H1 is supported.

#### Co-creation signals and sense of self-determination

*t*-Test results showed that two types of co-creation signals made observers feel significant differences in self-determination. Observers who received consumer self-designed co-creation signals had a higher sense of self-determination (*M*_designed_ = 5.212, *M*_produced_ = 4.491, *t* = −11.022, *p* < 0.05), autonomy (*M*_designed_ = 5.390, *M*_produced_ = 4.283, *t* = −14.558, *p* < 0.05), competence (*M*_designed_ = 4.985, *M*_produced_ = 4.504, *t* = −6.398, *p* < 0.05) and belonging (*M*_designed_ = 5.261, *M*_produced_ = 4.686, *t* = −7.689, *p* < 0.05), H2, H2a, H2b, and H2c are supported.

#### The mediating effect of self-determination

We used the PROCESS macro (model 4) by Hayes (2017) to test the mediation effect (Table 2). A bootstrap analysis revealed that the mediating effect of both self-determination and its sub-dimensions was significant. The mediating effect of self-determination was 0.781 (CI = [0.620, 0.957], excluding 0), and after adding self-determination, the direct effect was still significant (CI = [0.126, 0.290], excluding 0), indicating that self-determination played a partial mediation role. The mediating effect of sense of autonomy was 0.888 (CI = [0.719, 1.079], excluding 0). After adding a sense of autonomy, the direct effect was no longer significant (CI = [−0.041, 0.242], including 0), indicating that the sense of autonomy played a complete mediation role. The mediating effect of sense of competence was 0.386 (CI = [0.268, 0.521], excluding 0). The direct effect was still significant after adding a sense of competence (CI = [0.501, 0.704], excluding 0), indicating that competence played a partial mediation role. The mediating effect of sense of belonging was 0.473 (CI = [0.333, 0.628], excluding 0). The direct effect was still significant after adding a sense of belonging (CI = [0.410, 0.621], excluding 0), indicating that belonging played a partial mediation role. H3, H3a, H3b, and H3c are supported.

**Table 2:** Mediating effects of self-determination and its sub-dimensions.

Intermediate variables	Type of effect	effect	se	t	p	LLCI	ULCI
Sense of self-determination	Direct effects	0.208	0.042	5.006	0.000	0.126	0.290
	Indirect effects	0.781	0.086	–	–	0.620	0.957
Sense of autonomy	Direct effects	0.100	0.072	1.401	0.163	−0.041	0.242
	Indirect effects	0.888	0.091	–	–	0.719	1.079
Sense of competence	Direct effects	0.603	0.052	11.707	0.000	0.501	0.704
	Indirect effects	0.386	0.064	–	–	0.268	0.521
Sense of belonging	Direct effects	0.515	0.053	9.651	0.000	0.410	0.621
	Indirect effects	0.473	0.076	–	–	0.333	0.628

## Study 2: The moderating effect of mental simulation

### Experiment design

The primary purpose of experiment 2 was to test the moderating effect of mental simulation on the relationship between co-creation signals and sense of self-determination. The experiment used inter-group design 2 (self-produced co-creation signals vs. self-designed co-creation signals)*2 (process simulation vs. outcome simulation). The experimental process randomly divided subjects into four groups conveyed different types of co-creation signals to each group and initiated different kinds of mental simulations. The rest of experiment 2 was consistent with experiment 1. The initiation method of mental simulation referred to previous studies. After conveying the co-creation signal to the subjects, a specific situation was presented and guided to a corresponding mental simulation. The process simulation group drove the subjects to imagine the processes and steps that must be implemented to participate in co-creation. For example, what support will the enterprise provide in the co-creation process? What tools will be used? In contrast, the outcome simulation group leads the subjects to imagine the results and impact of co-creation. For instance, what abilities can be exercised after participating in co-creation? How do you feel after participating in co-creation? It then asked subjects to write down text from the questions and answers generated during the mental simulation.

To test the manipulation effectiveness of mental simulations, we carried out two tasks: degree of subjects’ engagement and scorer reliability. First, when the manipulation task of the mental simulation was finished, subjects’ engagement degree was measured through the question “To what extent are you involved in the above scenario simulation,” on a five-point scale, with higher scores indicating greater engagement. In addition, after the experiment, two scorers have been invited to code the imaginary situations written by subjects and calculated the scorer reliability by referring to the scoring methods of existing studies (Han and Wang, 2012). The experiment recruited 334 participants at a large public university in central China in early October 2021, with 298 valid subjects (112 male/186 female; mean age = 19.409 years old).

### Experiment results

#### Scale reliability

The Cronbach’s *α* for the sense of autonomy, sense of competence and sense of belonging, respectively, were 0.792, 0.756 and 0.759, all higher than 0.7, which all reached the reliability standard required by statistics.

#### Maneuverability test

The manipulative test of co-creation signal type showed that subjects could effectively determine the difference between self-designed co-creation signal and self-produced co-creation signal (*M*_designed_ = 5.807, *M*_produced_ = 2.095, *t* = −45.925, *p* < 0.05), independent variable manipulation therefore succeeded. The manipulation effectiveness of the moderating variable mental simulation tests included two tasks: degree of subjects’ engagement and scorer reliability. Subjects’ engagement measurements suggested that all participants devoted themselves to the experiment relatively well (*M* = 4.275). Scorer reliability invited two scorers to code the mental simulation text written by the participants and refer to the scoring methods of the existing research: in the process simulation group, if the participant’s text included information such as some plans for co-creation, rational thinking and other specific processes to achieve the goal, one point was awarded for each primary point involved. In the outcome simulation group, if the participant’s text included information such as emotion, evaluation and feelings after participating in co-creation, one point was awarded for each primary point involved. The results showed that the scorer reliability of the process simulation group was 0.912, *p* < 0.01, and the scorer reliability of the outcome simulation group was 0.940, *p* < 0.01. So the manipulation of the mental simulation was successful.

#### The moderating effect of mental simulation

**Figure 2:**
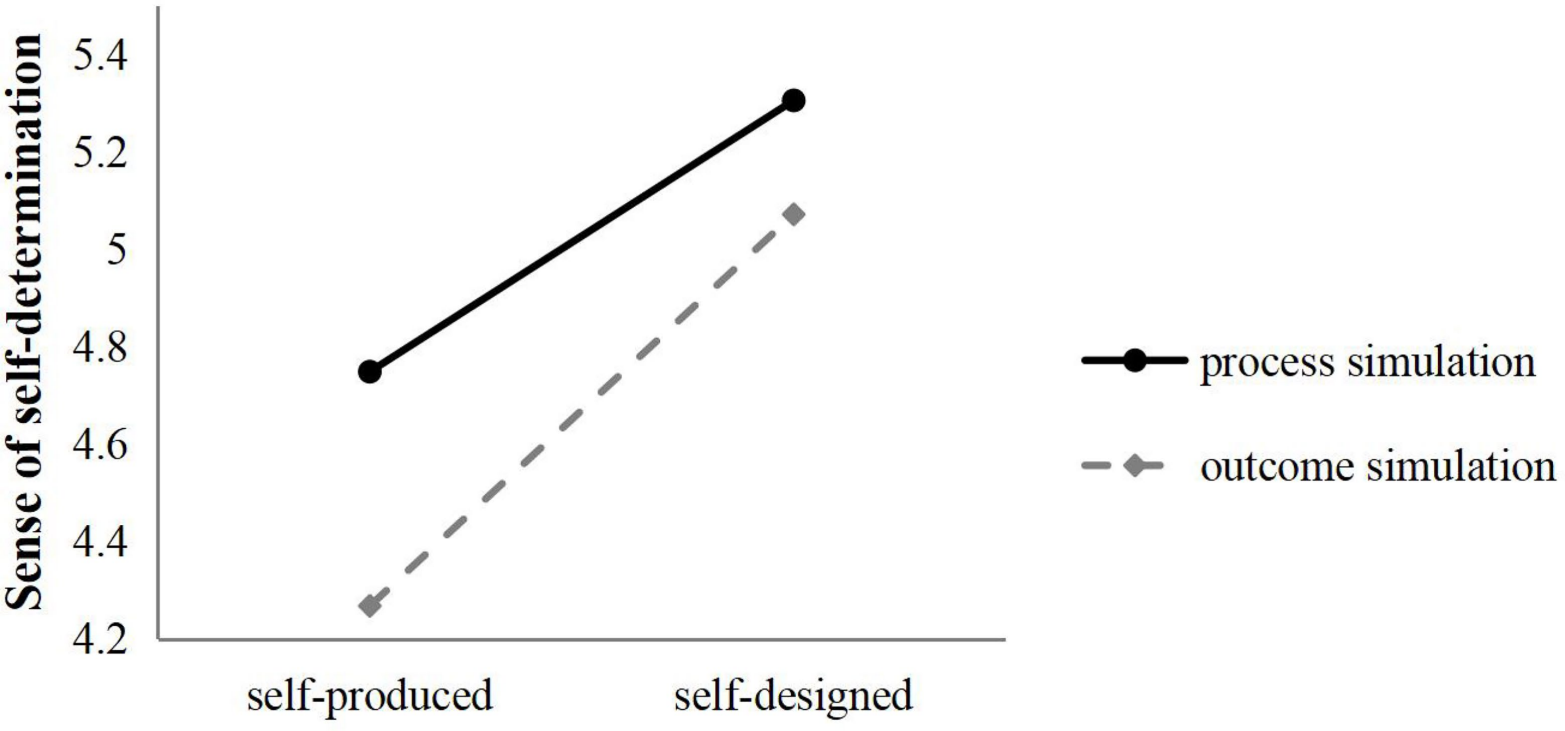
Moderating effect of mental simulation (sense of self-determination).

Two-factor ANOVA results revealed a significant interaction between the type of co-creation signal and the type of mental simulation in terms of sense of self-determination (*F*(1,294) = 11.231, *p* < 0.05), it indicated that after receiving the self-produced co-creation signal, observers who performed process simulation had a higher sense of self-determination than outcome simulation (*M*_process_ = 4.749, *M*_outcome_ = 4.268). After receiving the self-produced co-creation signal, compared with the outcome simulation, the process simulation also made observers have a higher sense of self-determination (*M*_process_ = 5.306, *M*_outcome_ = 5.072; Figure 2). The main effect of the co-creation signal (*F*(1,294) = 340.160, *p* < 0.05) and mental simulation (*F*(1,294) = 93.722, *p* < 0.05) was also significant, so mental simulation had a moderating effect on feelings of self-determination, and H4 is confirmed.

**Figure 3:**
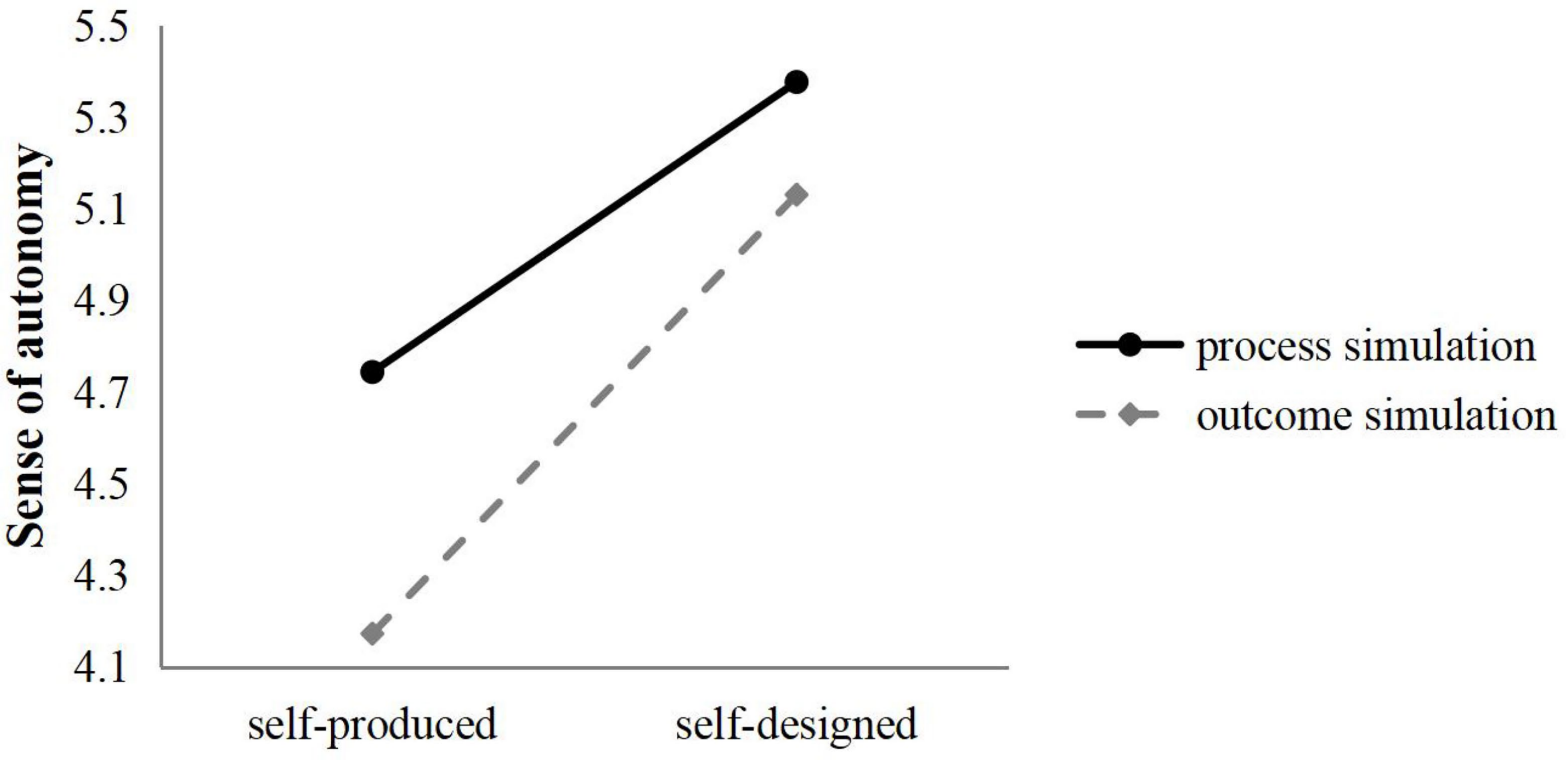
Moderating effect of mental simulation (sense of autonomy).

**Figure 4:**
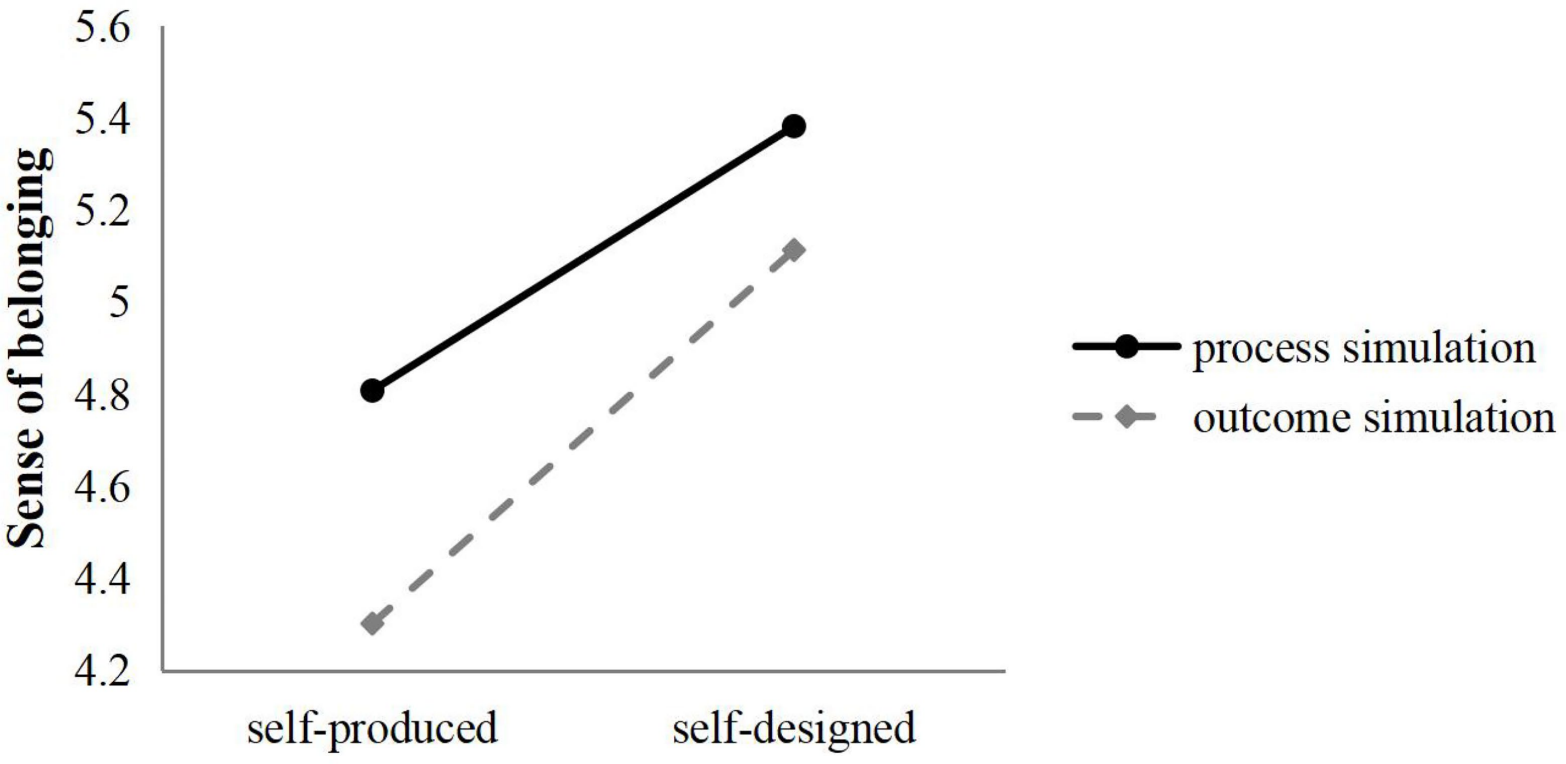
Moderating effect of mental simulation (sense of belonging).

Further analysis of the moderating effect of mental simulation on self-determination sense sub-dimension showed that in terms of autonomy and belonging sense, co-creation signal type had significant interaction with mental simulation type (sense of autonomy: *F*(1,294) = 8.847, *p* < 0.05; the sense of belonging: *F*(1,294) = 4.613, *p* < 0.05), indicating that after receiving the consumer self-production co-creation signal, compared with the outcome simulation, observers who conducted process simulation had a higher sense of autonomy (*M*_process_ = 4.744, *M*_outcome_ = 4.173) and belonging (*M*_process_ = 4.808, *M*_outcome_ = 4.302). After receiving the self-designed co-creation signal, the process simulation also gave observers a higher sense of autonomy (*M*_process_ = 5.377, *M*_outcome_ = 5.131) and a greater sense of belonging (*M*_process_ = 5.382, *M*_outcome_ = 5.113; Figures 3, 4). The main effects of co-creation signal (sense of autonomy: *F*(1,294) = 212.585, *p* < 0.05; sense of belonging: *F*(1,294) = 157.198, *p* < 0.05) and mental simulation (sense of autonomy: *F*(1,294) = 56.186, *p* < 0.05; sense of belonging: *F*(1,294) = 49.305, *p* < 0.05) were significant, H4a and H4c are effectively supported. For the sense of competence, the interaction between co-creation signal type and mental simulation type was not significant (*F*(1,294) = 2.664, *p* = 0.104 > 0.05), indicating that there was no moderating effect of mental simulation type between co-creative signal and sense of competence, H4b is not supported.

## Conclusion and discussion

### Conclusion

Based on self-determination theory, we investigated the mechanism of different types of co-creation signals on observers’ co-creation willingness and examined the moderating effect of mental simulation. The findings show that co-creation signals delivered by enterprises positively influence observers’ co-creation willingness, and self-designed co-creation signals lead to stronger co-creation willingness than self-produced co-creation signals. According to self-determination theory, participation in co-creation conforms to the observers’ inherent tendency to pursue self-development and self-growth, and enterprises’ initiative to release co-creation signals indicates an excellent external environment for observers to participate in co-creation.

After fully considering the support of the external environment and their own internal needs, observers believe that participation in co-creation can satisfy their basic psychological needs and thus generate a strong sense of determination. That is reflected in the improvement of the sense of autonomy, sense of competence, and sense of belonging, and enhances the observers’ intention to co-create. In addition, different types of co-creation signals make observers feel significant differences in self-determination sense. Observers who received self-designed co-creation signals felt a greater sense of self-determination than those who received self-produced co-creation signals—a greater sense of autonomy, competence, and belonging leads to a stronger willingness to co-create.

Further, our research finds that mental simulation moderates co-creation signals and a sense of self-determination. Specifically, observers who perform process simulation after receiving co-creation signals have a stronger sense of autonomy and belonging than outcome simulation. However, there are differences in the dimension of sense of competence, as observers are consumers who have not yet participated in co-creation. They lack rich co-creation experience and clear cognition about the co-creation process and result. Therefore, when they receive co-creation signals, it is not easy for them to differentially perceive the effectiveness of their behavior and whether it can achieve the expected goal of co-creation based on different types of mental simulation. In both process and outcome simulations, observers who received self-designed co-creation signals perceived a stronger sense of self-determination than self-produced co-creation signals. It is embodied in three dimensions the importance of autonomy, competence and belonging.

### Theoretical implications

The theoretical implication of this study is reflected in three aspects. Firstly, the discussion is carried out from the perspective of co-creation observers, which enriches and improves the research on the outcome effect of co-creation. Most co-creation studies focus on consumers and enterprises that have already participated in co-creation. The motivation of participation, interactive process, content, and mechanism of co-creation has been thoroughly discussed (Andreu et al., 2010; Fuchs and Schreier, 2011; Kao et al., 2016). Only a few pieces of literature explore the influence of consumer participation in co-creation signals on co-creation observers. Compared with a few consumers who have participated in co-creation, more consumers are observers of value co-creation. Therefore, it is necessary to study the results and effects of co-creation from the perspective of co-creation observers.

Secondly, the research of co-creation signals is extended and deepened by exploring the influence mechanism and effect difference of co-creation signal types from self-determination. The few studies on the effect of co-creation signals have focused on observers’ interpretation of co-creation signals regarding the identity similarity between observers and participating clients (Fuchs et al., 2013; Thompson and Malaviya, 2013). In addition, proper research has mainly discussed the transmission and effects of co-creation signals that empower consumers to “design” (Dahl et al., 2015; Kristal et al., 2016). This study analyzes the influence mechanism of co-creation signal on observers’ co-creation willingness from the self-determination perspective, therefore, more complete and comprehensive. At the same time, based on the form of consumer participation in co-creation, the study also explores the signaling effects of empowering consumers to “self-design” and “self-produce” on observers of co-creation, which further supplemented the co-creation signal research.

Thirdly, our research deepens the boundary research of the co-creation signal effect and expands the research field of mental simulation. It also enriches mental simulation research in marketing communication and extends it to the practice of value co-creation. Based on the consideration of co-creation observers’ characteristics, mental simulation is selected as a moderating variable to explore its moderating role between co-creation signals and their impact effects, which is more relevant and appropriate.

### Managerial implications

This study provides enterprises implications for using co-creation signals effectively and optimizing co-creation marketing practices. Firstly, companies should expand their co-creation focus to co-creation observers and recognize the importance of actively portraying co-creation signals. Co-creation practices should not only concern consumers who have already participated in co-creation. More important is to attract those observers who have not yet been involved in co-creation to participate in co-creation. Observers are the most extensive market components, who need to be effectively guided and motivated to actively participate in co-creation and contribute to the enterprise’s growth. In addition, companies should recognize that co-creating signals are an effective way for companies to promote themselves and influence consumers’ perception of them. Enterprises can convey their customer-centered business philosophy and other relevant critical information to consumers by actively using co-creation signals. This can enhance consumers’ recognition and trust in enterprises. In marketing communication, companies can use the signal of co-creation to tell a good story, highlight the powerful interaction of customers in co-creation, and promote co-creation development. In this way, it is of great significance to promote brand communication, strengthen the relationship between consumer enterprises and shape differentiated competitive advantages.

Secondly, the effectiveness of co-creation signals should be enhanced by optimizing the co-creation material and its presentation. Enterprises need to carefully design all aspects of consumer participation in co-creation activities to make it the material source of co-creation signals. Companies should emphasize participants’ autonomy in co-creation activities when issuing co-creation signals. At the same time, it should also highlight participants’ talent and ability in co-creation, strengthen the connection between participants and observers, and promote the interaction between consumers and enterprises in the process of co-creation. By displaying the supporting elements such as co-creation platforms, service information, and emotional incentives provided by companies, the enterprise expands information sources of consumers’ interpretation of co-creation signals, enhances their sense of self-determination, and generates a solid willingness to participate in co-creation. It is essential to ensure that consumers are involved in all aspects of the co-creation process under a reasonable arrangement. As conditions permit, co-creation signals can be used more widely to attract consumers to design co-creation and increase their sense of autonomy, competence, and belonging, encouraging them to continue participating in co-creation.

Finally, mental simulation can be skillfully used for marketing communication to strengthen consumers’ perception of co-creation and actively guide them to participate in co-creation. Companies can explore concrete ways to co-creation signals transmission. For example, consumer co-creation activities can be distilled into juicy and valuable co-creation stories. Enterprises can use graphics, short films and other forms to guide consumers to imagine the process and results of co-creation. It can help consumers better learn and understand co-creation activities and obtain information about customer orientations the interactivity and autonomy of co-creation, which will attract consumers to participate in the co-creation. When transmitting co-creation signals and using mental simulation strategies to communicate with consumers, businesses can share as much detail as possible in the co-creation signals about the specific tasks and the process of co-creation activities. It can effectively guide consumers to carry out process simulation while telling a good co-creation story, strengthening their cognition of co-creation, and leading them to participate in co-creation actively.

### Limitations and future research

This study has several limitations that could be addressed in future studies. First, this study adopts the situational experiment method, which has good internal validity. However, it may lead to insufficient external validity as the experiments are not conducted in a natural signaling environment. In the future, the experimental situation can be furthered realistically through a combination of text, pictures, videos and AI, and the signal transmission scene can be created as close as possible to reality. Besides, it can use mixed research designs, methods such as self-reported and secondary data can be used to expand data sources and repeatedly verify the validity of conclusions, which will improve the external validity of the research.

Second, this study selects T-shirts as the experimental stimulus, and the sample is limited to university students. The effectiveness of co-creation signals may vary depending on the product types and the signal receiving group. Therefore, future studies can consider including a larger and heterogeneous population as recipients of co-creation signals and use different types of products as the stimulus, examining similarities and differences in the effects of co-creation signals to enrich co-creation research.

Third, in this study, the name of enterprise and brand are concealed in the process of co-creation signals transmission, which can effectively prevent consumers from generating corresponding brand associations and interfering with the experimental results. But it may reduce the validity of the research. In the management practice of enterprises, factors such as brand awareness and brand reputation will impact the perceptions, attitudes and behaviors of consumers. Therefore, the brand is an influencing factor that cannot be ignored during the co-creation process between enterprises and consumers. Future studies can be combined with existing brands in the market, considering the impact of brand factors on the effectiveness of co-creation signals to promote relevant research.

## Data availability statement

The raw data supporting the conclusions of this article will be made available by the authors, without undue reservation.

## Author contributions

LZ and NZ contributed to the framework and theoretical model of the study. NZ organized the database, performed the statistical analysis, and wrote the first draft of the manuscript. LZ and HW contributed to manuscript revision. All authors contributed to the article and approved the submitted version.

## Conflict of interest

The authors declare that the research was conducted in the absence of any commercial or financial relationships that could be construed as a potential conflict of interest.

## Publisher’s note

All claims expressed in this article are solely those of the authors and do not necessarily represent those of their affiliated organizations, or those of the publisher, the editors and the reviewers. Any product that may be evaluated in this article, or claim that may be made by its manufacturer, is not guaranteed or endorsed by the publisher.
